# Design and validation of a low-cost open-source impedance based quartz crystal microbalance for electrochemical research

**DOI:** 10.1016/j.ohx.2022.e00374

**Published:** 2022-11-08

**Authors:** Rens J. Horst, Antonis Katzourakis, Bastian T. Mei, Sissi de Beer

**Affiliations:** a Sustainable Polymer Chemistry Group, Department of Molecules & Materials, MESA+ Institute for Nanotechnology, University of Twente, 7500 AE Enschede, The Netherlands; b Photocatalytic Synthesis Group, Department of Molecules & Materials, MESA+ Institute for Nanotechnology, University of Twente, 7500 AE Enschede, The Netherlands; c Super B Lithium Batteries, Hengelo, The Netherlands

**Keywords:** Electrochemical Quartz Crystal Microbalance, Electrochemistry, Open-source

## Abstract

The quartz crystal microbalance (QCM) measurement technique is utilized in a broad variety of scientific fields and applications, where surface and interfacial processes are relevant. However, the costs of purchasing QCMs is typically high, which has limited its employment in education as well as by scientists in developing countries. In this article, we present an open-source QCM, built on the OpenQCM project, and using an impedance-based measurement technique (QCM-I), which can be built for <200 euro. Our QCM allows for simultaneous monitoring of the frequency change and dissipation, such that both soft and rigid materials can be characterized. In addition, our QCM measurements can be combined with simultaneous electrochemical measurement techniques (EQCM-I). We demonstrate the validity of our system by characterizing the electrodeposition of a rigid metallic film (Cu) and by the electropolymerization of aniline. Finally, we discuss potential improvements to our system.

## Graphical abstract

**Figure:**
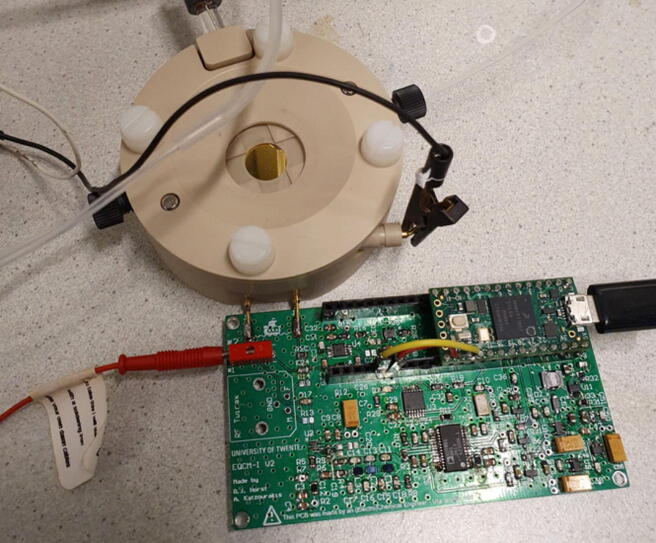

Specifications table

**Table:** 

**Hardware name**	Impedance-based Electrochemical Quartz Crystal Microbalance
**Subject area**	• Engineering and material science
	• Electrochemical Surface Science
**Hardware type**	• Electroacoustic gravimetry
	• Electrochemical Assays
**Closest commercial analog**	Gamry eQCM 10 M, QSense Explorer, OpenQCM Q-1
**Open source license**	Creative Commons Attribution-ShareAlike 4.0 International License (CC BY-SA 4.0)
**Cost of hardware**	€200
**Source file repository**	https://osf.io/q6yk4/ or https://doi.org/10.17605/OSF.IO/PR8EW

## 1 Hardware in context

### 1.1 Introduction

Since its conception in 1959 by Sauerbrey [1], the quartz crystal microbalance has become a widely adopted technique in surface science research [2]. Initially, QCMs were mostly used as thin film deposition monitors to be able to control the film thickness. However, pioneering work by Nomura and Okuhara published in 1982 extended the applicability of the technique to liquid based systems [3]. Though, application of Sauerbrey’s theory to liquid systems proved problematic as it assumed that the mass should be rigidly adsorbed, while a liquid phase adds viscoelastic contributions to the resonance response. This prompted theoretical development related to the viscoelastic contributions of materials on a QCM by Kanazawa and Gordon [4], eventually extending the use of a QCM into the soft matter domain [5], [6]. Nowadays, QCMs are still used to monitor layer deposition but its realm of applications extends far beyond that to for example sensors [7] and bioanalytics [8], [9], [10], liquid viscometry [11] and the study of polymeric thin films [12]. The viscoelastic properties of these soft matter systems can be assessed by the use of the more modern QCM-D or QCM-I capable of simultaneous frequency and dissipation measurements [13].

The possibility of measuring apparent mass changes at the solid–liquid interface also sparked the development of the electrochemical quartz crystal microbalance (EQCM) for the field of interfacial electrochemistry [14]. Here, one of the quartz electrodes is in contact with the electrolyte and serves as both quartz crystal oscillator electrode and the working electrode (WE) of an electrochemical cell. Careful consideration of signal grounding allows for the combination of electrochemical techniques and QCM operation, offering new insights into interfacial electrochemical processes. Additionally, the use of QCMs with dissipation monitoring allows to study emerging applications in greater detail. Reported applications range from metal electrodeposition [15], [16] and leaching studies [17], ion dynamics [18], porous solids characterization [19], organic electropolymerization [20], [21] to for example monitoring self assembled monolayer formation [22]. The versatility of (E) QCM measurements lends it very well to combinations with other complementary techniques like in situ spectroscopic ellipsometry [23], surface plasmon resonance [24], [25], scanning probe microscopy [26], [27] or Fourier-transform infrared spectroscopy [28], [29].

While EQCMs have been shown to be very powerful tools for surface science, current commercial systems can be expensive and inflexible. Many labs employ home-built setups consisting of tracking generators and frequency counters or more expensive lock-in amplifiers and network analyzers. In an effort to avoid the use of costly or complicated setups, researchers of Novaetech S.r.l. have come up with an open-source QCM-I project, OpenQCM. By leveraging the fast-paced advancements in integrated circuits over the past decades a relatively inexpensive and easy to use system capable of frequency and dissipation measurements was designed.

In this paper we will present and validate an open-source EQCM-I design that builds upon the foundations of the OpenQCM project and allows researchers to simultaneously measure mass changes and electrochemical responses. The main goal of this paper is to present a possible next step towards an open-source EQCM-I. Our design uses a similar impedance based measurement principle and uses the same signal generator chip. The main difference between the two projects is that our device can be used together with electrochemical measurement techniques. Adding to that, our quartz crystal is driven by a high speed operational amplifier which yields a more stable current source than the architecture used in the OpenQCM project. Furthermore, our design uses a single-ended measurement chip with a ∼30 dB higher dynamic range and 4 times faster voltage output rise time than the more complex differential-mode OpenQCM chip. This in principle enables our device of measuring faster and with a higher resolution. Additionally, the device has a programmable driving amplitude nearly twice that of the OpenQCM device enabling higher measurement resolution in damping liquid media. The small footprint and relatively inexpensive nature (∼€ 200) compared to the more expensive OpenQCM systems (€ 1500) make it suited for incorporation into all sorts of measurement setups. Moreover, aside from very precise frequency measurements at up to 70 MHz, it is capable of assessing dissipation in liquid environments during electrochemical experiments within an adequate time resolution. The device is demonstrated using two electrochemical cases: one employing electrodeposition of a rigid metallic film and one with electropolymerization forming a thin polymeric film to demonstrate both the frequency response and dissipation. In the end, some opportunities are identified to further improve the design.

### 1.2 Theory

As proper understanding of quartz crystal microbalance (QCM) operation is not trivial, we deem it important to provide a theoretical section on its general operation and application to more complex liquid based electrochemical systems. This theoretical framework is not meant as an exhaustive body of literature, but merely as a guide to point out critical pieces of information about QCM operation which a potential user should be aware of. For more in-depth information we refer to the references provided in the manuscript as well as the elaborate review by Buttry and Ward [14] and the book by Johannsmann [30].

#### 1.2.1 Quartz crystal microbalance basics

A QCM is a highly sensitive mass sensor, which consists of a thin quartz substrate sandwiched between two vapor-deposited electrodes driven by an oscillator circuit. The resonance frequency of this quartz crystal oscillator (typically 5–10 MHz) is highly susceptible to the addition of mass onto the electrodes. A mass change at the surface induces a shift in the resonance frequency (Δf) and consequently enables the detection of sub-monolayer weight changes (e.g. 0.1% of a Au monolayer) [31]. Compared to an electronic mass balance with a resolution of 0.1μg a QCM (5 MHz) can be more than a hundred times more sensitive [32].

A QCM owes its high inherent sensitivity to the stability of the oscillation and the advent of high resolution sub-Hertz frequency measurements [33]. Furthermore, its ease of use and capability to work at room temperature and at atmospheric pressure, which is more an exception than a rule for surface science techniques, attribute to its popularity in thin film studies and soft matter research. Up to 2019 it was estimated that there have already been more than 14.000 publications focussed on QCM applications alone [34].

The operation of the QCM is based on the piezoelectric property of quartz, discovered by Jacques and Pierre Curie in 1880. When mechanical stress is applied to crystals with a noncentrosymmetric space group (e.g. quartz or tourmaline) an electrical potential is generated over the crystal proportional to the magnitude of the mechanical stress. Shortly thereafter, the converse piezoelectric effect was also demonstrated where the application of an electronic potential over these crystals resulted in a proportional mechanical strain [35].

**Fig. 1:**
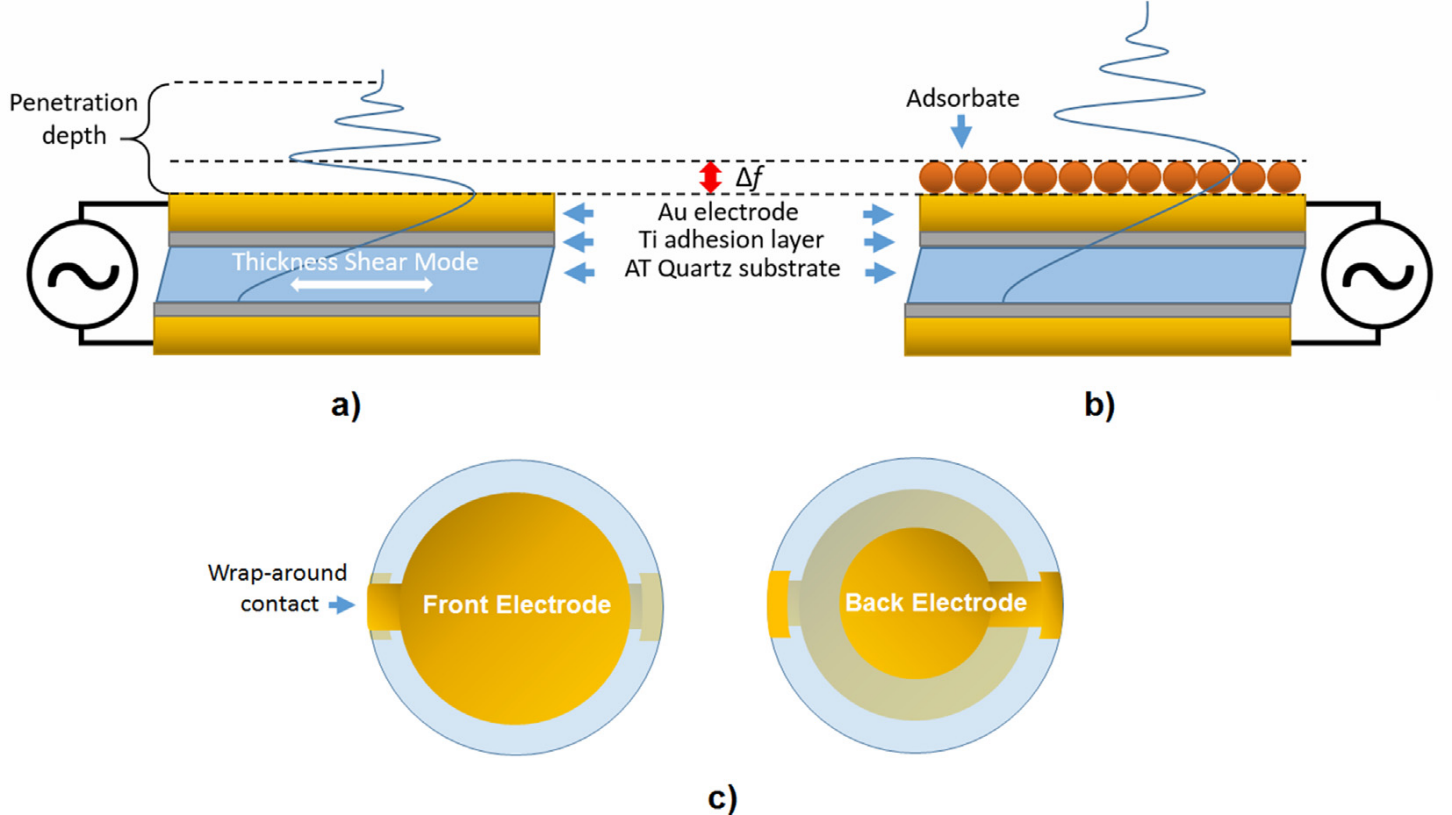
Schematic cross-section of a quartz crystal showing thickness sheer oscillation overlaid with the fundamental resonance wave (a), the change in oscillation resonance frequency (Δf) when a rigid mass adsorbs to the surface (b) and front and back view of a quartz crystal with wrap-around electrode typically used for liquid measurements (c).

This converse piezoelectric effect is the foundation of the operating principle of the QCM. In 1959 Sauerbrey [1] first discovered that tracking the resonance frequency of the converse piezoelectric effect could be used to measure mass changes on the quartz surface. Quartz crystals sandwiched between two vapor-deposited metallic electrodes could be excited by applying an alternating electric field across the crystal. This produces an elastic deformation causing a vibrational motion of the crystal. Crystal symmetry, the orientation and frequency of the electric field and the crystal cut, at what crystallographic plane it was cut, determine the extent and nature of this electromechanical coupling. While for example, SC-cut [36] crystals oscillate well in gasses their mode of oscillation precludes their use in liquids. AT-cut [37] crystals however, oscillate in thickness shear mode making them suitable for use in vacuum, gas and liquid [30]. Fig. 1a shows such a crystal and its fundamental oscillation mode.

AT-cut crystals further owe their popularity to their superior temperature stability as compared to other quartz crystal cuts [37]. AT-cut quartz crystals have the inflection point of their temperature dependent resonance frequency around room temperature. This makes their operation relatively temperature insensitive compared to other types of quartz crystals when operating at room temperature [38]. Still, it is important to carefully control the temperature as the resonance frequency can still drift at a rate of 1ppm/K, which would mean a frequency shift of 5Hz/K^−1^ for a 5 MHz crystal. A stable temperature is therefore a very important attribute of accurate QCM measurements.

The acoustic wave has a certain penetration depth dependent on the measurement medium, e.g. in water this is typically 250 nm for a 5 MHz crystal. Sauerbrey theorized that any mass that would be rigidly attached to the quartz surface would simply increase the resonator thickness and thus lower the resonance frequency of the acoustic wave (depicted in Fig. 1b). By assuming that the added mass possesses the same acoustic properties as quartz Sauerbrey came to the famous equation that is often represented as Eq. 1.

(1) $$\Delta m_{f}=-\frac{\sqrt{\rho_{q}G_{q}}}{2f_{0}^{2}}\frac{\Delta f}{n}=-C_{f}\frac{\Delta f}{n}$$

Here $\Delta m_{f}$ is the change in areal mass density and Δf is the change in resonance frequency from the reference frequency $f_{0}$. The quartz density is $\rho_{q},G_{q}$ is the shear modulus of the crystal (often represented as $\mu_{q}$ in literature). $C_{f}$ is the mass sensitivity constant, which for a standard 5 MHz crystal corresponds to 17.7ng cm^−2^Hz^−1^. In Eq. 1, *n* denotes and odd number specifying the overtone, the fundamental frequency corresponds to n=1. As a result of the electronic characteristics of the quartz crystal only odd overtones can be excited.

In general, measurements at higher frequencies offer a higher sensitivity as the frequency shift is larger for the same areal mass density increase. For example, a 10 MHz crystal has a $C_{f}$ of 4.4ng cm^−2^Hz^−1^ compared to 17.7ng cm^−2^Hz^−1^ for a 5 MHz crystal. A downside of using crystals with higher fundamental frequencies is that they are more prone to fracturing as they are thinner. Additionally, while measuring at higher overtones also yields a higher frequency sensitivity, a downside is that the oscillation is often much weaker than the fundamental resonance which could be a problem in a highly damping environment. Furthermore, it is important to note that the Sauerbrey equation only holds under certain assumptions: (1) the mass has to be rigidly attached to the crystal surface, (2) the measurement environment it was derived for is air not liquids and (3) the attached mass should not be too large (< 2%) for the small load approximation used in the derivation of the equation.

When the work of Nomura and Okuhara [3] showed QCM measurements in liquids the need arose for new theory on the contribution of this viscous medium to the resonance response. Fig. 1c shows a typical crystal used for liquid measurements with the electrical contacts on one side. Work published in 1985 [4] demonstrated the development of a mathematical model called the Kanazawa-Gordon equation after its inventors (Eq. 2).

(2) $$\Delta f_{\mathrm{KG}}=-f_{0}^{\frac{3}{2}}\sqrt{\frac{\eta_{l}\rho_{l}}{\pi \rho_{q}G_{q}}}$$

**Fig. 2:**
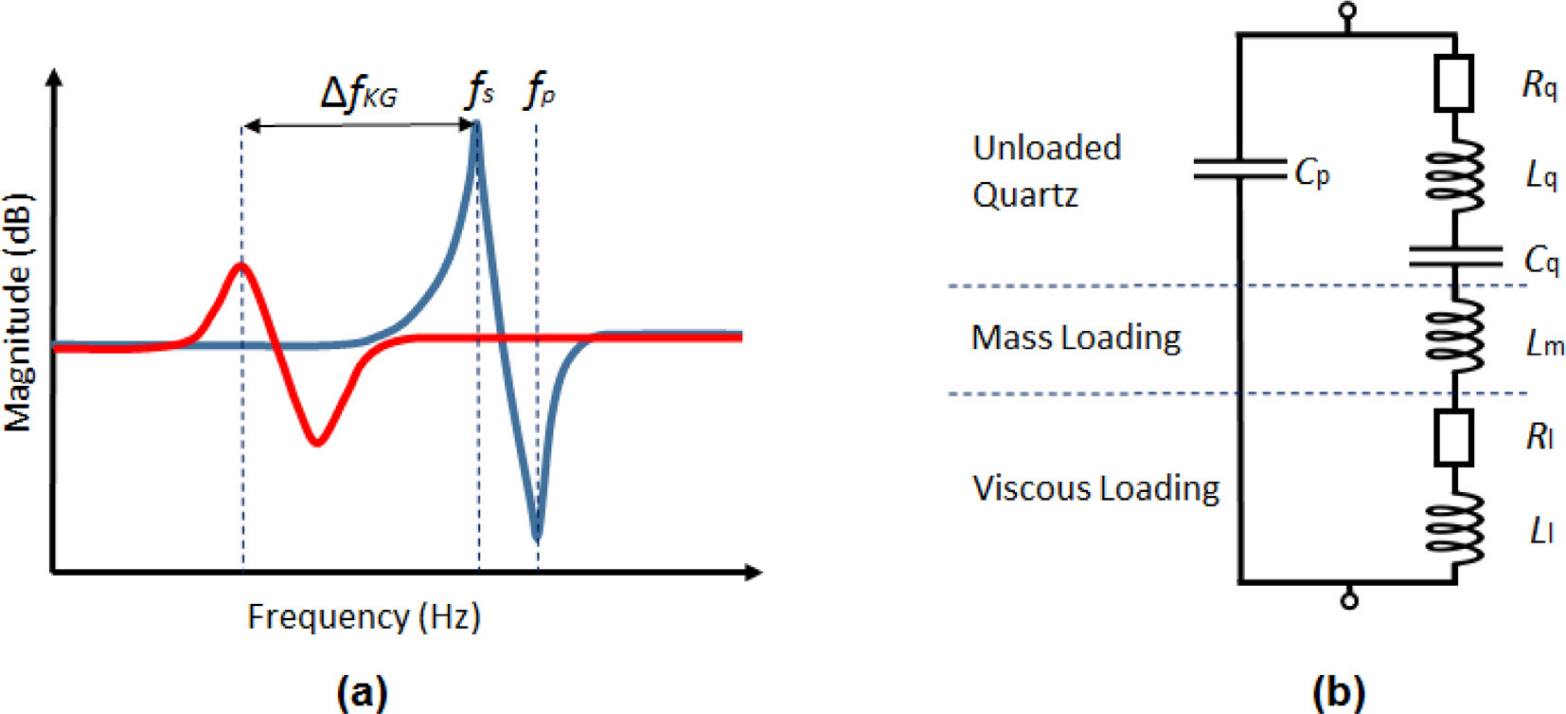
(a) Shows the difference of measuring in air (blue) or a viscous liquid (red) on the quartz admittance plot. Viscous media incur a negative frequency shift ($\Delta f_{\mathrm{KG}}$) on the resonance frequency and also dampen the magnitude of the oscillation. (b) Is a schematic of the BVD equivalent circuit of a QCM under both viscous and mass loading.

It implies an added negative frequency shift ($\Delta f_{\mathrm{KG}}$) as a result of the viscous nature of the liquid with absolute viscosity ($\eta_{l}$) and density ($\rho_{l}$). The Kanazawa-Gordon shift can be illustrated with an admittance plot comparing resonance in air and water as depicted in Fig. 2a. The curve of the response in liquid is shifted with respect to the response curve in air. For pure water and a 5 MHz crystal this shift would be approximately 0.7 kHz. Furthermore, the viscous liquid dampens the magnitude of the oscillation thereby increasing the so-called dissipation (*D*), often represented as the change in dissipation (ΔD). Dissipation is the reciprocal of the crystal quality factor (*Q*) which defines the sharpness of the resonance response. *D* can be used to characterize the viscoelastic behaviour of adsorbed systems like bio-molecules or polymeric films.

The dissipation value can be determined by (1) a ring-down technique where the oscillation is allowed to decay and the decay envelope is fitted to a decaying sinusoid or (2) by characterization of the full width at half maximum of an impedance spectrum. The ring-down technique is used in the more complicated QCM-D (D of decay) and the width determination is used in QCM-I/A (Impedance or Admittance) systems. The ring-down technique makes the QCM-D generally faster as impedance techniques require lengthy frequency sweeps to characterize the resonance curve, especially in liquid systems.

The resonance response of a QCM is often explained in terms of a Butterworth-Van Dyke (BVD) equivalent circuit [39] (see Fig. 2b). In this model $R_{q},L_{q}$ and $C_{q}$ are the motional resistance, inductance and capacitance respectively. Their mechanical analogs would be energy dissipation or loss, inertia and an elastic component. The inductance (inertial) and capacitance explain the existence of the series resonance ($f_{s}$ in Fig. 2a) for unloaded quartz. At $f_{s}$ the terms $L_{q}$ and $C_{q}$ are equal and only the small $R_{q}$ term is left. The parallel resonance ($f_{p}$) is caused by static capacitance of the electrodes and is a high impedance resonance mode. Addition of a rigid mass is said to only add an inductive term $L_{m}$ and merely shifts $f_{s}$ and $f_{p}$ negatively. If the added mass would also induce an appreciable viscous damping a resistive term should be included as well. The viscous liquid loading contributes both an inductive ($L_{l}$) term responsible for the $\Delta f_{\mathrm{KG}}$ shift and resistive ($R_{l}$) term as vibrational energy is dissipated into the liquid.

**Fig. 3:**
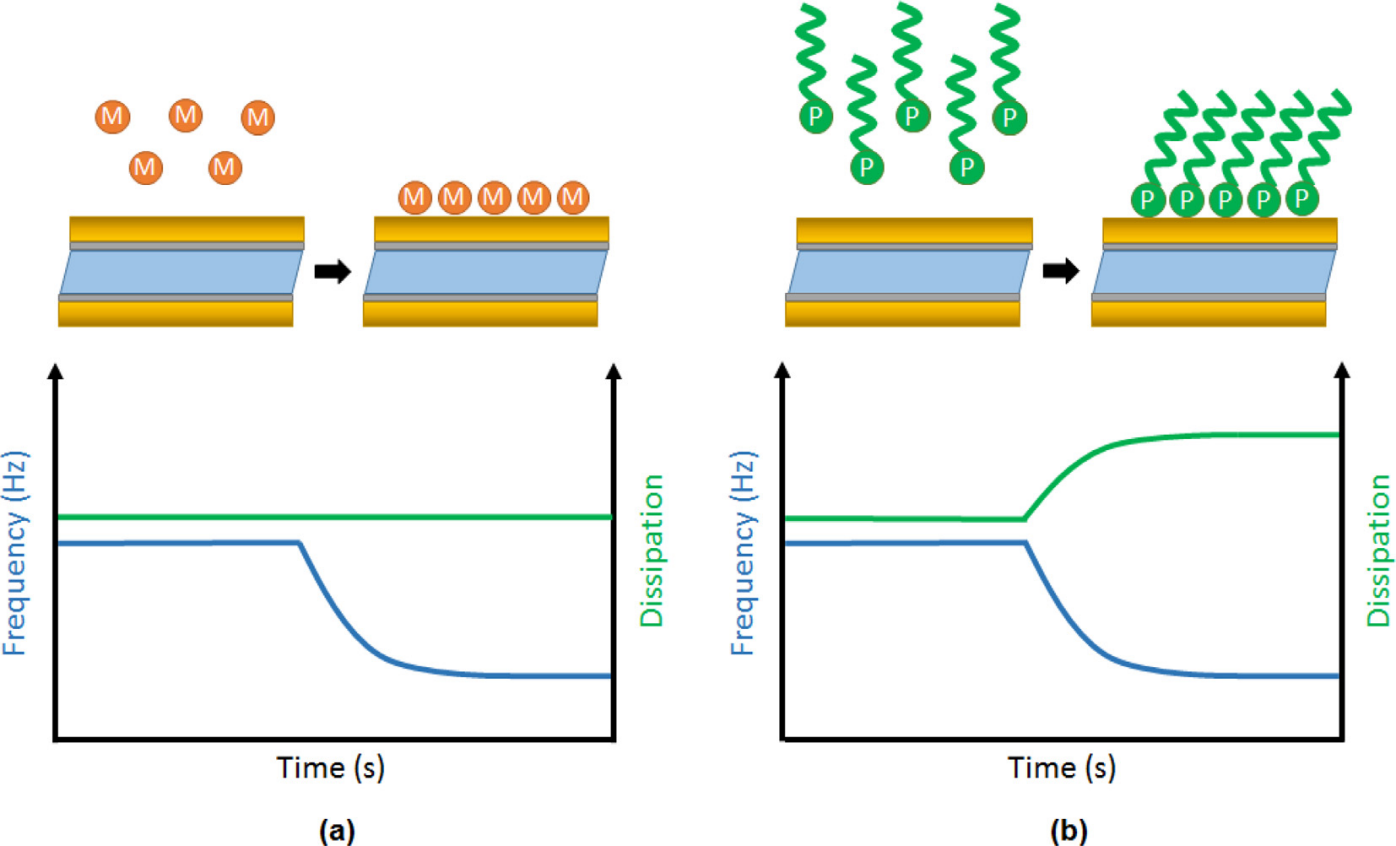
(a) Shows the frequency and dissipation response to the adsorption of a rigid metallic layer (M), while (b) shows the adsorption of a viscoelastic polymer (P) film and its effect on the resonance frequency and dissipation.

While the adsorption of a rigid layer of metal or a viscoelastic polymeric film cause a negative frequency shift as a result of the inertial loading of the quartz oscillation, a clear difference can be observed in their influence on the measured dissipation. The addition of the rigid metallic layer, as seen in Fig. 3a, should in principle not significantly change the oscillation energy dissipation as it is tightly coupled to the oscillating surface. Adsorption of the viscoelastic polymer film onto the vibrating surface does however add to the dissipation of oscillatory energy and is thus said to increase the dissipation.

It should be noted that the analysis of thick viscoelastic films can deviate from the expected frequency response. At a certain thickness Δf and ΔD might start showing a non-linear response, so that for example Δf no longer changes proportionally to the actual mass that is added [40]. This phenomenon can be detected by measuring at several overtones as this will cause Δf/n and ΔD/n to diverge. By using the Voigt model the layer thickness can still be estimated based on the overtone data, but an interested reader is referred to the work of Dunér et al. for more information on this topic [40].

#### 1.2.2 Electrochemical quartz crystal microbalance

Electrochemistry, which is mostly confined to liquid electrolytes, was quickly combined with QCM [15] and QCM-D/I/A (EQCM) and proved to be a very powerful set of complementary techniques. EQCM provides insight into mass and structural changes at electrode surfaces related to electronic processes, e.g. ion intercalation, corrosion, electrodeposition or electropolymerization. An EQCM should be calibrated with a well known redox couple, e.g. Cu(II) + 2$e^{-}$ ⇌ Cu(0), by measuring the slope of Δf vs. ΔQ (See Eq. 3) in electrodeposition. The calibration factor $C_{f}$ is based on the combination of mass and charge balance of the electrodeposition and is analogous to the calibration factor used in Eq. 1. Here ΔQ is the cumulative charge transferred, *F* is Faraday’s constant, *n* the number of electrons transferred per elementary reaction, $M_{w}$ the molar weight and the $\times 10^{9}$ factor is for converting to ng.

(3) $$\frac{1}{C_{f}}=\frac{\Delta (\Delta f)}{\Delta (\Delta Q)}\frac{\mathrm{nF}}{M_{w}\times 10^{9}}$$

**Fig. 4:**
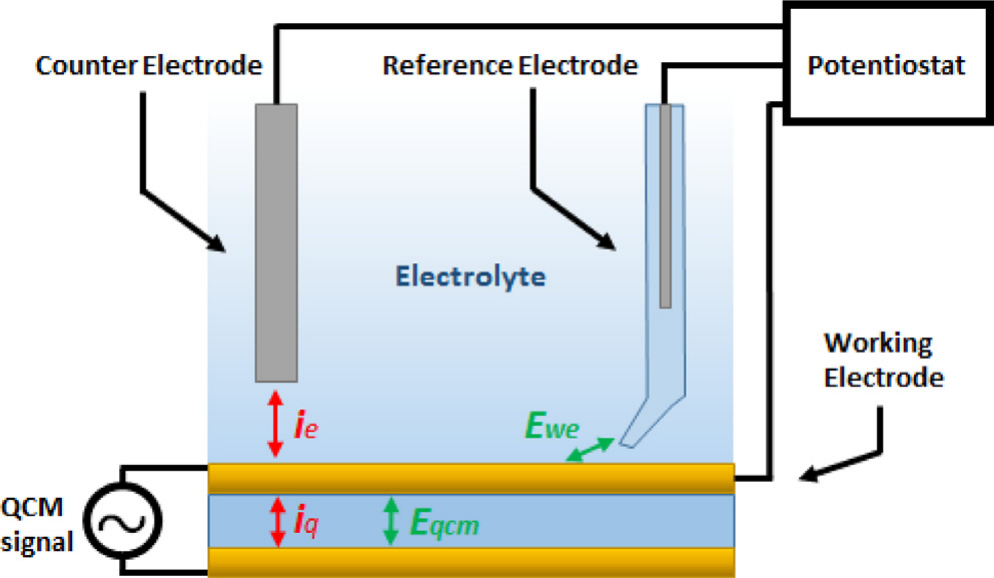
Schematic representation of typical floating EQCM configuration with a three electrode cell. The currents $i_{e}$ and $i_{q}$ are the respective electrochemical current as controlled by the potentiostat and QCM oscillator.

To successfully combine the QCM with electrochemical techniques it is important to understand how a potentiostat used for electrochemistry functions in a three electrode cell. The potentiostat is used to set the potential, $E_{\mathrm{we}}$, of the working electrode (WE) with respect to a well-defined and stable reference electrode (RE). Stability of the RE is of paramount importance in electrochemistry and that is why the potentiostat only allows for a minute current to run between WE and RE to not affect the potential. Instead, it controls $E_{\mathrm{we}}$ by forcing more or less current ($i_{e}$ in Fig. 4) between the WE and the counter electrode (CE) to influence the electronic state of the WE.

Now, the key factor for an EQCM-I to work is that the potentiostat should be floating and completely isolated with respect to the EQCM-I voltage. So, the EQCM-I controls the voltage ($E_{\mathrm{qcm}}$) relative to an isolated ground via its internal circuitry and is the origin of current $i_{q}$ in Fig. 4. While the potentiostat controls $E_{\mathrm{we}}$ on top of $E_{\mathrm{qcm}}$ relative to a floating RE with the current $i_{e}$ running through electrolyte. By virtue of their isolated grounds the QCM and potentiostat signal cannot interfere and allow for simultaneous measurements.

## 2 Hardware description

**Fig. 5:**
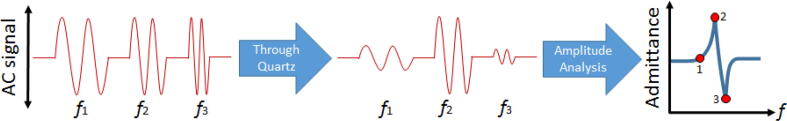
Simplified drawing of an impedance based measurement principle where the quartz impedance is a function of signal frequency. Here $f_{2}=f_{s}$ (low impedance) and $f_{3}=f_{p}$ (high impedance).

For the design, an impedance based measurement approach was chosen because of its capability to measure both Δf and *D* while possessing a relatively uncomplicated architecture. As depicted in Fig. 5, an EQCM-I measures the effect of passing a certain range of discrete frequencies with equal amplitude through the quartz crystal. As a result some frequencies will be damped slightly ($f_{1}$), one maximally damped ($f_{3}$) at $f_{p}$ and one will resonate at $f_{s}$ and be minimally damped ($f_{2}$). The amplitude of each frequency is recorded and forms an admittance plot (reciprocal of impedance) and from this the resonance frequency $f_{s}$ and the dissipation *D* can be determined as described in Section 1.2.1. Continuous frequency sweeps allow for monitoring changes in frequency and dissipation over time.

**Fig. 6:**
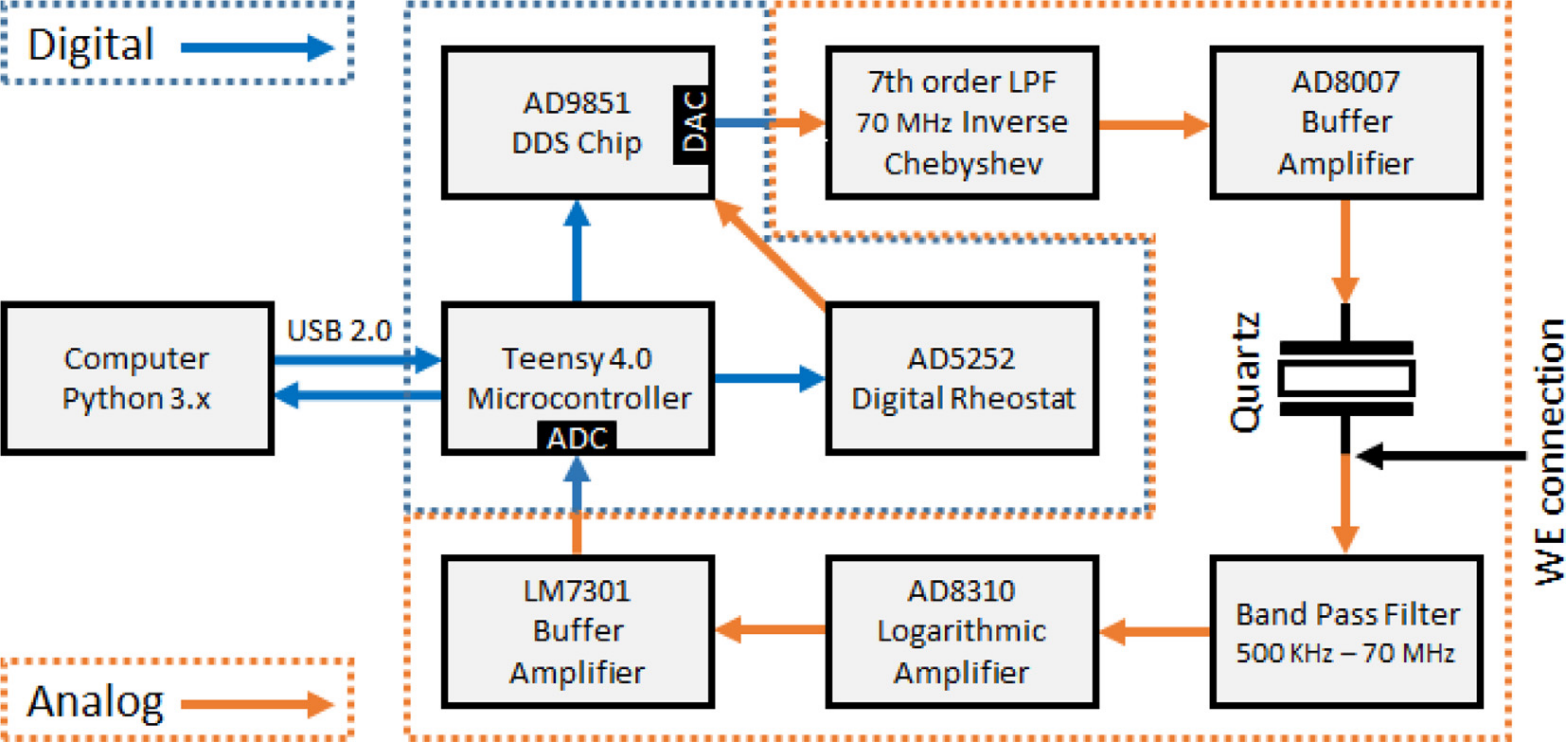
Block diagram of the most critical parts of the EQCM-I design presented in this manuscript. The diagram is divided into a digital and analog region while at the crossover regions analog-to-digital (ADC) or digital-to-analog (DAC) conversions take place. The electrochemical working electrode (WE) connection is also indicated.

A block diagram overview of the proposed design can be seen in Fig. 6. At the heart of the device is a Teensy 4.0 microcontroller (MCU) clocked at 600 MHz, which can be easily programmed using the Arduino IDE with the Teensyduino extension. It is used to receive instructions from a computer running a Python 3 data acquisition (DAQ) script (SI page 12) over serial USB (2.0). Once sweep instructions are received by the MCU, it programs the AD9851 direct digital synthesis (DDS) chip to output a sine wave of the first specified frequency. The DDS chip has a high resolution output frequency of 0.04 Hz potentially enabling the device to measure sub-nanogram changes [2]. The MCU programs the AD5252 rheostat to limit the output current of the DDS chip and thereby sets the sine wave amplitude. As a result of the DDS signal generation, higher order harmonics of the target frequency are also present in the output signal. For that reason, the signal is passed through a 70 MHz inverse Chebyshev low pass filter that is characterized by very low pass band ripple and strong attenuation because of its 7^th^ order architecture.

The signal is then buffered by a high speed AD8007 buffer amplifier to provide a high input impedance to the DDS output, so it takes over the current sourcing for the quartz oscillation. After the signal passes through the quartz that possesses a certain impedance for that frequency the attenuated signal is filtered through a band pass filter (BPF) to remove any unwanted noise that falls outside of the frequency domain of interest. This is done to ensure that the AD8310 logarithmic amplifier receives a low noise signal. The AD8310 is a high speed, high dynamic range logarithmic amplifier, which makes it well-suited for the frequency measurement range of interest. As the AD8310 is used in single-ended mode it measures the envelope of the signal and outputs a DC voltage that is proportional to the amplitude of the sine wave. This DC voltage is buffered by an LM7301 unity gain amplifier and read by the two 12-bit ADCs of the MCU with a maximum sample rate of ∼1 MS/s. The reading for each frequency is averaged over 4096 ADC conversions to allow for a high amplitude measurement accuracy.

In general each frequency sweep has a span of 10 kHz with a frequency step of 10 Hz in order to characterize the quartz frequency response. After the MCU has acquired all the data for one sweep it transmits this over serial USB to the PC running the Python DAQ script. The data is processed and interpolated using a spline function to ascertain *D* and $f_{s}$ at a sub-Hz resolution. The reason for using spline interpolation stems from the gain in acquisition time resolution as running through each sweep at sub-Hz resolution results in slow measurements. When the processing is complete new sweep instructions are sent to the MCU and this process repeats until the measurement is stopped.

In the design stage a lot of lessons learned by the developers of OpenQCM project could be used. Their design incorporated the same signal generator DDS chip as our design. While our design shares its impedance-based design with the OpenQCM project, there are some notable differences. First, the low pass filter (LPF) design was changed to an inverse Chebyshev type as this allows for higher quality pass band as it only features stop band ripple. This was very important to the use of the AD8310 with ∼30 dB higher dynamic range and faster voltage output rise time than the AD8302 of the OpenQCM project, which should improve measurement speed and resolution.

Furthermore, the use of a common emitter and common collector amplifier cascade buffer by OpenQCM was discarded as the use of the high speed AD8007 buffer amplifier should in theory outperform the cascade. Especially, at high frequency measurements since it has a greater gain bandwidth which improves the buffer feedback and thus should improve signal integrity and measurement stability.

A further difference is that our device has been designed to work simultaneously with electrochemical experiments so that the two measurement techniques do not interfere. This was achieved by proper grounding of the signals, effectively isolating the two signals from each other. The QCM and electrochemical signals are said to be ’floating’ with respect to each other. This has the added benefit of being able to electrochemically validate the quartz mass sensitivity using a highly reversible redox couple as the deposition/stripping charge can be correlated to a change in mass.

Aside from the hardware differences, the design offers for the first time the ability to perform simultaneous electrochemical as well as mass balance measurements for a fraction of the costs of commercial systems. The small footprint of the device also allows for the incorporation of the device in other measurement setups to combine with different techniques. The open-source nature of the project and recommendations presented in this publication also allow others to further improve on the current design. The device can be used in various fields of research and we list a few examples below.

1. ▪ Quantitative study of the deposition or growth of materials using electrochemical techniques, but can also be used to monitor detachment or dissolution of material. Examples would be electrodeposition or electrostripping of metals from a surface, electropolymerization on an electrode.
2. ▪ Quantitative study of the change of materials like the oxidation or corrosion of metals, like studying the process of platinum oxidation and subsequent dissolution which is a major problem in Pt-electrocatalysis.
3. ▪ Quantitative study of the capture and release of ions for organic or inorganic battery or capacitor electrode materials or for the development of redox-active sensors.
4. ▪ Aside from simultaneous measurements the device can also be used for studying electrochemical changes and mass changes separately where one might be interested in electrochemical growth of a material and assess its stability over time by monitoring mass loss without applying an electrochemical potential.

## 3 Design files summary

All the design files as listed below can either be found at the specified OSF project URL in the table or at the OSF registry by accessing the following URL: https://doi.org/10.17605/OSF.IO/PR8EW.

**Table:** 

**Design filename**	**File type**	**Open source license**	**Location of the file**
*EQCM-I Bill of Materials*	*Excel spreadsheet*	*GPL-3.0*	https://osf.io/q6yk4/
*EQCM-I Schematic*	*PDF file*	*GPL-3.0*	https://osf.io/q6yk4/
*Project Folder*	*ZIP folder*	*GPL-3.0*	https://osf.io/q6yk4/
*Project Outputs for EQCM-I*	*RAR folder*	*GPL-3.0*	https://osf.io/q6yk4/
*Python DAQ*	*Python file*	*GPL-3.0*	https://osf.io/q6yk4/
*Python DAQ Script PDF*	*PDF file*	*GPL-3.0*	https://osf.io/q6yk4/
*Teensy Firmware*	*INO file*	*GPL-3.0*	https://osf.io/q6yk4/
*Teensy Firmware Script PDF*	*PDF file*	*GPL-3.0*	https://osf.io/q6yk4/

*EQCM-I Bill of Materials:* *Excel spreadsheet containing the bill of materials for the PCB components. All component types as ordered are reported, except for one inductor which was only available with a different inductance 560nH vs 510nH. Use this list during soldering and use the designator column as reference.*

*EQCM-I Schematic:* *PDF overview of the electronic circuit design used for the device. The comment in red refers to a decoupling capacitor that was missed in the design, but could be inserted in a superfluous resistor footprint (R17).*

*Project Folder:* *Folder that contains the entire Altium designer project. Since Altium designer is proprietary software it cannot be easily opened, but free trials are available. This folder is required when changes to the board are required, but one could also start from the schematic in a freely available software.*

*Project Output for EQCM-I:* *Gerber files required for direct production of the PCB. These files can be sent to any PCB manufacturer and should allow them to produce the board.*

*Python DAQ:* *Python script used for the data acquisition. Care should be taken in using the right libraries to get the software to work.*

*Python DAQ Script PDF**:* PDF version of Python DAQ script for quick access.

*Teensy Firmware:* *Arduino IDE (.INO) file containing the Teensy 4.0 firmware that should be programmed onto the microcontroller board.*

*Teensy Firmware Script PDF:* *PDF version of Teensy Firmware for quick access.*

## 4 Bill of materials summary

.

**Table:** 

**Designator**	**Component**	**Number**	**Cost per unit - EUR**	**Total cost - EUR**	**Source of materials**	**Material type**
*EQCM-I Bill of Materials*	*PCB components*	*1*	*140 EUR*	*140 EUR*	*Ordered from Digikey.com*	*Electronic components*
*Project Output for EQCM-I*	*Printed circuit board*	*1*	*8 EUR*	*8 EUR*	*Ordered from JLCPCB.com*	*Printed circuit board*
*Teensy 4.0*	*Microcontroller board*	*1*	*26 EUR*	*26 EUR*	*Ordered from TinyTronics.nl*	*Microcontroller*
*Cable*	*Micro USB cable*	*1*	*2 EUR*	*2 EUR*	*Electronic hardware store*	*Connection cable*

**TOTAL**	…	…	…	**176 EUR**	…	…

It is assumed that people who will attempt to build the device at least have access to basic electronics tools like a soldering iron, consumables, a multimeter, etc.

## 5 Build instructions

As the device operates in the lower end of the RF frequency spectrum ground vias are added as a precaution to avoid excessively large ground loops. Grounding and wiring of high frequency signals should be considered carefully. In case changes to the board lay out are required please refrain from changing the footprints and wiring of all components marked with U. Nevertheless, if changes to these parts are necessary we refer to the respective data sheets of the components for reference designs.

**Preparation** & **Tips**

1. ▪ For soldering the relatively small 0806 packages a soldering iron with a small tip is preferred.
2. ▪ For the chips the use of a hot air station would be advised, but it is not strictly necessary.
3. ▪ A 60 MHz oscilloscope could also come in useful in characterization of the signals and power lines.
4. ▪ An anti-static band is also recommended when working with the ICs.
5. ▪ The gerber design files as available on the OSF repository can be sent directly to the PCB manufacturer. It should be noted that the board comprises 4 layers. For our design we used FR-4 dielectric material, 0.5 oz copper and HASL surface finish.
6. ▪ Ordering of the components can be done based on the bill of materials. It is wise to order some spare components as you might drop and lose a component once in a while.
7. ▪ Once the orders come in it is wise to check them for completeness and sort them based on the components specifications (e.g. resistors with resistors).
8. ▪ Be sure to have plenty of soldering consumables before starting.
9. ▪ Before soldering components to the board it is important to apply solder to the respective pads.
10. ▪ As the board makes use of extensive ground planes (heat sinks), it can sometimes be difficult for the solder to flow properly. Preheating of the ground pads with the solder iron can help with this.

**Building the device**

1. ▪ Step 1: Mount the PCB in a holder or lay it down flat on a bench.
2. ▪ Step 2: Start with the components marked U. Components are clearly labeled (Fig. 7) by either an R (resistor), C (capacitor), U (IC chip), L (inductor), D (diode), X (crystal), L (header) followed by a number. This number is linked to the detailed bill of materials which specifies which specific component value should be used.

**Fig. 7:**
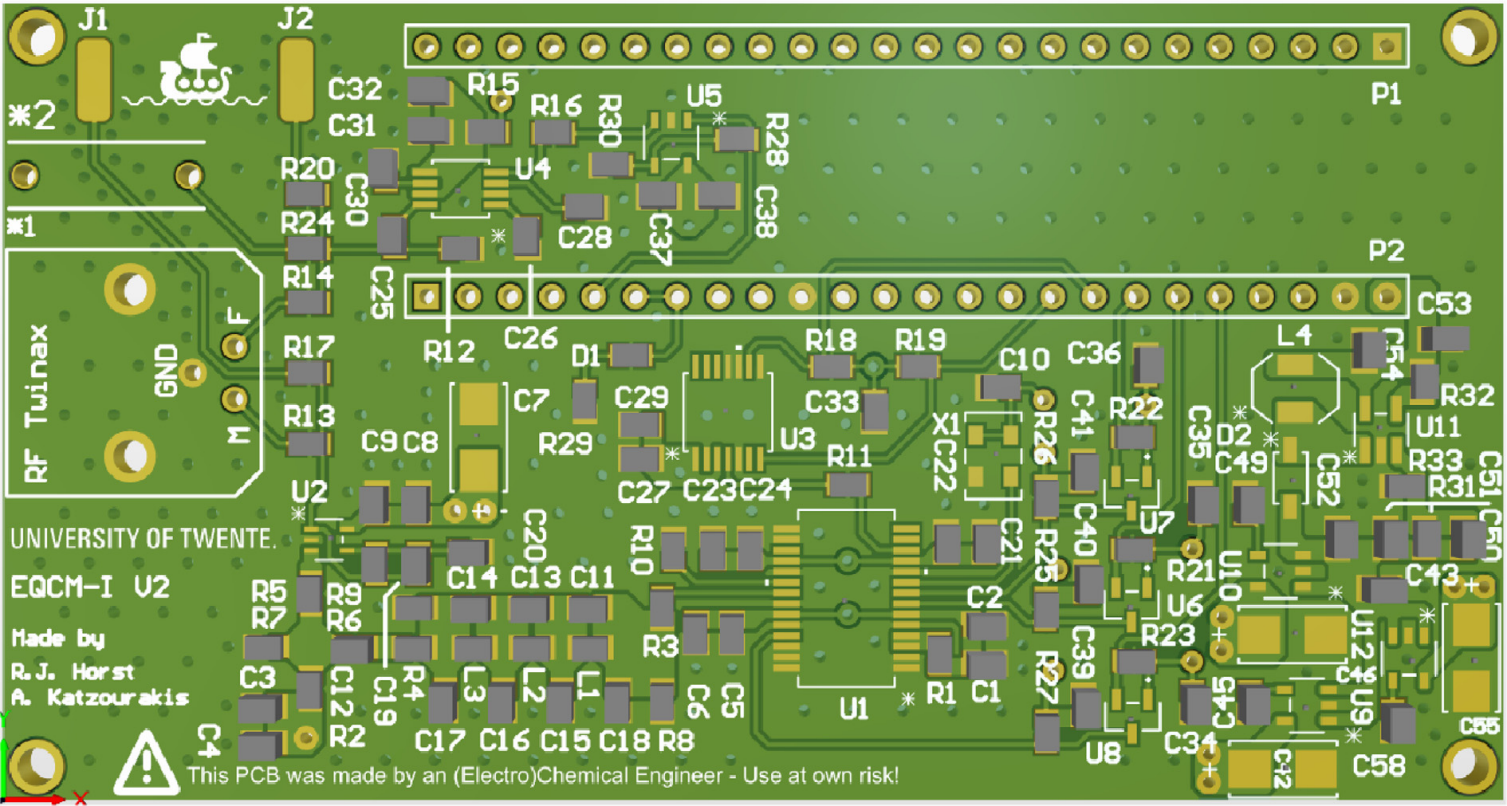
Top view of the PCB for the EQCM-I showing the component indicators in silk screen (white).
3. ▪ Step 3: After a soldering connection has been made it is important to check the contact resistance between the pad and the component by a simple multimeter continuity test. This is especially important for the IC packages featuring small pads like U1, U2 and U3. Improper solder connections can lead to a dysfunctional device.
4. ▪ Step 4: After all the components have been soldered, the Teensy 4.0 can be plugged into the headers P1 and P2 as far as possible to the right of the PCB. As the header footprint was designed for a Teensy 3.6, two jumper wires should be soldered to the Teensy 4.0. One on pin 16 and it should go into P2 slot 5 and one from pin 13 to P2 slot 6.
5. ▪ Step 5: Now the USB cable can be plugged in and it is important to verify the right  + 5 V supply voltages are present on the board by checking the voltage over C42, 43, 46 and 55.
6. ▪ Step 6: The Teensy firmware can now be uploaded to the board by using the Teensyduino IDE. If it is uploaded successfully the status LED (D1) should light up.
7. ▪ Step 7: To test the communication between the DAQ software and the Teensy 4.0 the Teensy firmware variable ’new_command’ can be changed to a value of 2.
8. ▪ Step 8: The output of the system can be probed between J1 and GND with an oscilloscope and a 5 MHz sine wave should now be visible. When this signal is observed the ’new_command’ variable in the firmware can be changed back to 1. The device should now be operational.
9. ▪ Step 9: There are several options in terms of connectors. We simply soldered the board through J1 and J2 to the cell as the cell itself did not provide any signal shielding, so using a shielded Twinax cable made no sense. Cells that do incorporate shielding should preferably be connected with a shielded cable.

## 6 Operation instructions

### 6.1 General

**Fig. 8:**
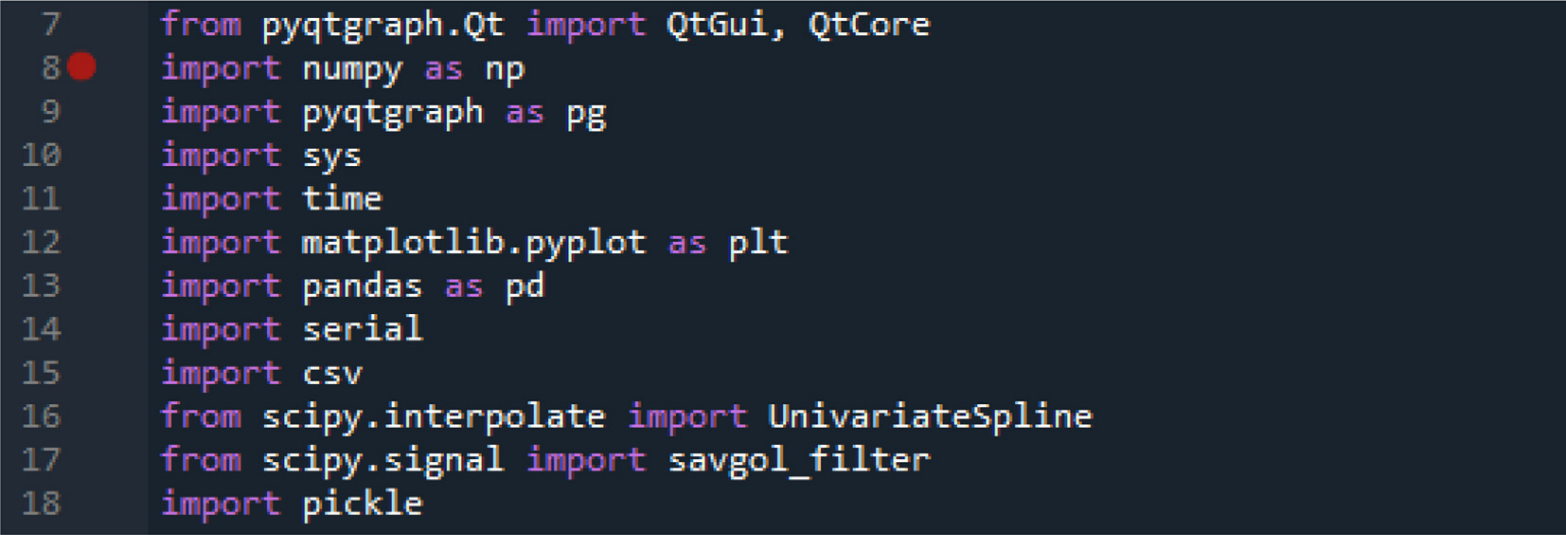
Code snippet showing the Python libraries required for the DAQ software.

**Fig. 12:**
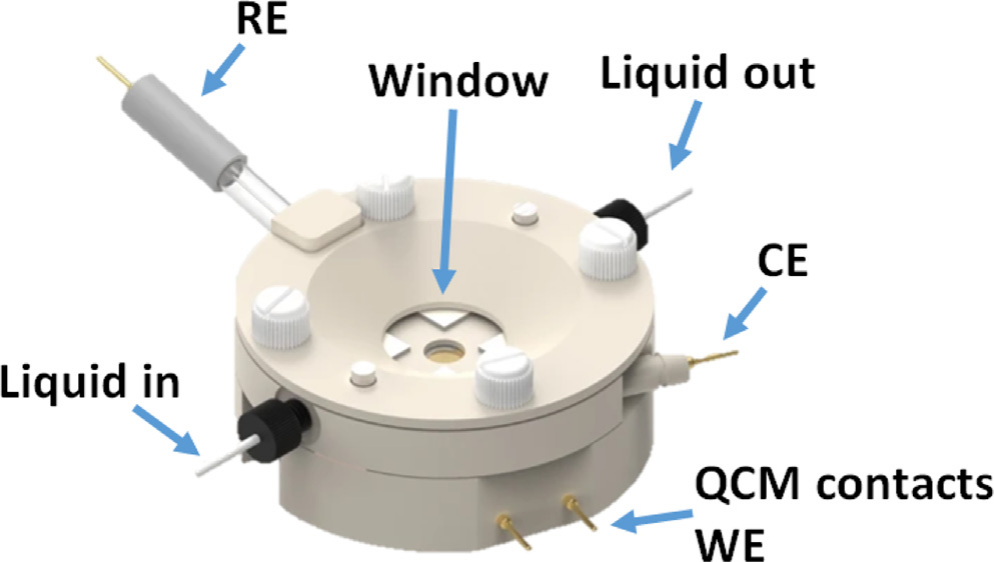
EQCM flow cell of the company Redox.me used in the experiments combining electrochemistry and QCM-I.

The device was used as constructed and was coupled to a measurement cell by soldering to the cell contacts. The particular cell used (Redox.me EQCM flow cell, see Fig. 12) could be combined with in situ Raman spectrometry but in principle any (home-made) EQCM cell could be used. The board features a possibility to connect using a BNC connector, a popular connector in the RF field, but any interested party can also modify the board to suit a different connector style. The Teensy 4.0 enables a simple USB connection to the PC running the DAQ software. To run the DAQ software a Python version 3.9 is required, we used Spyder as Python IDE. The Teensy MCU will need to be programmed by the user through USB and the Teensyduino IDE before the EQCM-I can be used. To run the DAQ software the libraries depicted in Fig. 8 are required.

**Fig. 9:**
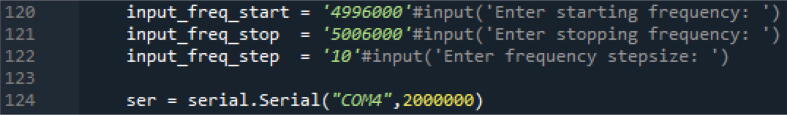
Code snippet showing the frequency sweep input section and the COM port selection line.

To be able to start a measurement it is important that the right COM port is selected in line 124 in order for the DAQ software to communicate with the EQCM-I during the measurement. The sweep parameters can be defined in lines 120–122. Take care that higher resolution sweeps take longer! Fig. 9,.

**Fig. 10:**
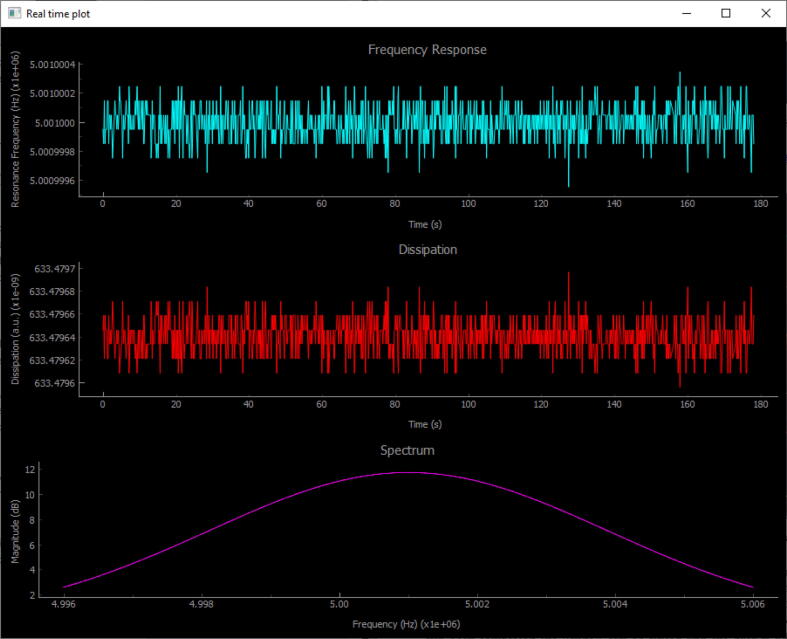
Screen capture of the measurement interface that appears when the DAQ software is running. Note that the data in this image was simulated just to show what the measurement interface looks like.

**Fig. 11:**
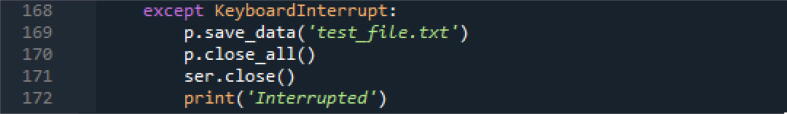
Code snippet showing the text file declaration line.

If the settings are properly chosen a screen like the one in Fig. 10, Fig. 11 should appear showing the measured frequency response, dissipation and a real time plot of the sweep result.

Sensor installation can differ per cell used, but it is important to take into consideration that the quartz crystals should be evenly compressed in order to not break and have a isotropically applied stress. The working electrode of an external potentiostat can be connected to the same pin as the top contact for the QCM measurements to allow for simultaneous electrochemical and gravimetric measurements. After a measurement has been completed the window can be exited and the data is automatically saved to a text file. The name of the text file (line 169) should be defined before starting the experiment. Otherwise the user risks overwriting existing data.

## 7 Validation and characterization

### 7.1 Materials

The chemicals H_2_SO_4_ (98%), HClO_4_ (70%), H_2_O_2_ (30%), Na_2_SO_4_ (99% anhydrous), CuSO_4_ (99% anhydrous), ethanol (technical grade) and aniline (99.5%) were obtained from Merck/Sigma–Aldrich and used as received. Demineralized water produced with a Merck Millipore Milli-Q system was used for all solutions. For all experiments 14 mm 5 MHz 330μm thick AT-cut quartz crystals with approximately 200 nm Au on a 10 nm Ti adhesive coating were used (Novaetech S.r.l.). It should be noted that the quartz crystals used to validate the device are also compatible with other commercial QCMs, allowing for cross-checking of the device performance as the type of quartz required merely depends on the cell geometry that is used and not on the device hardware. This means that the device should be able to use any quartz crystal available as long as its resonance frequency of interest does not exceed 70 MHz. Electrochemistry experiments were performed using an UD446 RE-1B Ag/AgCl 3M NaCl reference electrode from ALS. A Redox.me EQCM flow cell (see Fig. 12) was used equipped with a Pt counter electrode. The cell itself is made out of PEEK, FFKM O-rings and a quartz window and has a 2.2 mm electrolyte height which corresponds to an electrolyte volume of 4.5 mm.

### 7.2 Methods

Prior to each measurement the QCM crystals were cleaned in piranha-solution (3:1 of H_2_SO_4_: H_2_O_2_) to remove all organic contaminants. Care should be taken when working with piranha-solution as its strong oxidative cleaning capability renders it extremely dangerous. After piranha cleaning the crystals were rinsed with water, then extensively with ethanol and were finally dried under nitrogen. Before use solutions were purged extensively with nitrogen to remove dissolved oxygen. EQCM-I measurements were performed in a carefully rinsed (water) EQCM flow cell (Fig. 12) while using a peristaltic pump (10 mL min^−1^) to circulate the electrolyte. Operators should be careful to avoid pressure transients produced by the peristaltic pump reaching the quartz crystal. A simple solution is using sufficiently long tubing so that the transient is damped over the length of the tube. Electrochemical measurements were performed using a Biologic VMP3 Potentiostat running EC-lab software while EQCM-I measurements were conducted using the homemade EQCM-I device with a 10 kHz sweep range at 10 Hz resolution. Results were fitted to a cubic univariate spline to determine Δf and ΔD. Measurements were only started when the open cell potential ($E_{\mathrm{oc}}$), Δf and ΔD were sufficiently stable.

### 7.3 Results & discussion

**Fig. 13:**
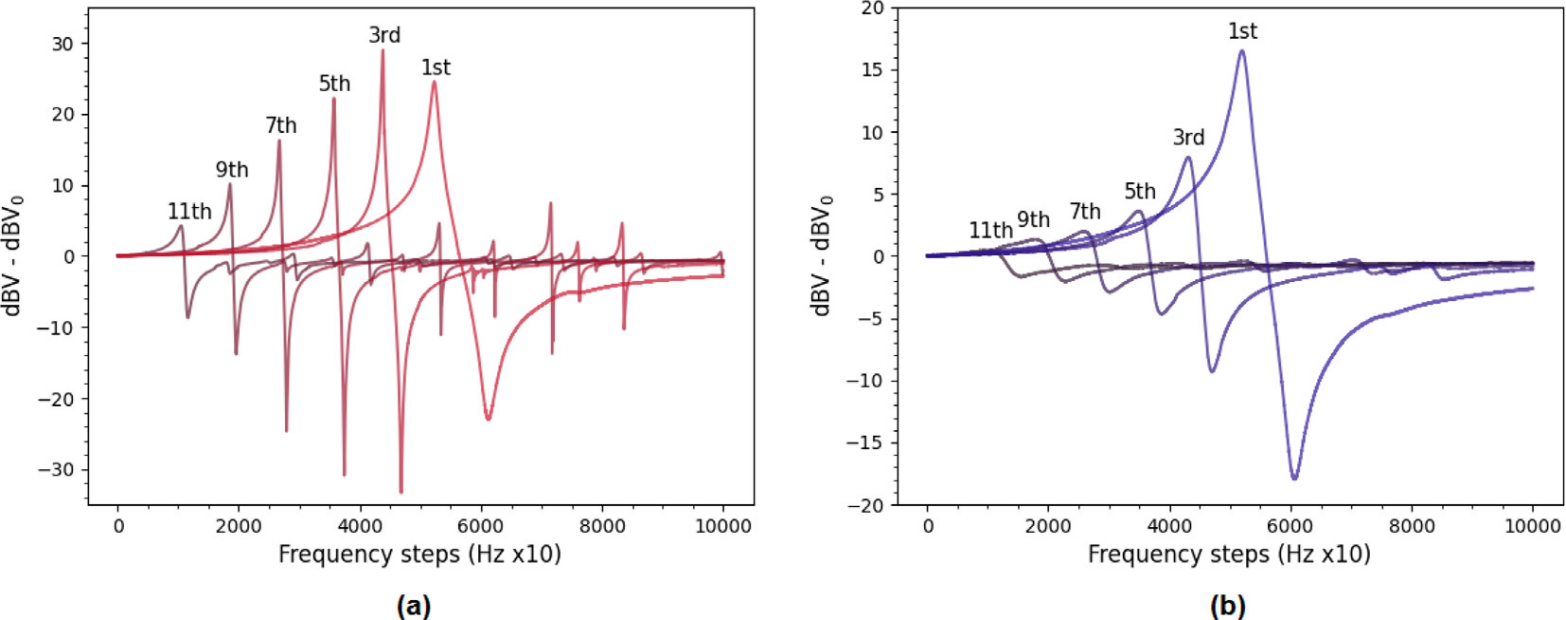
Admittance plots of frequency sweeps of the resonance response of a 5 MHz AT-cut quartz crystal in air (a) and water (b). The sweeps were recorded around multiples of 5 MHz over a range of 100 kHz with 10 steps.

**Fig. 14:**
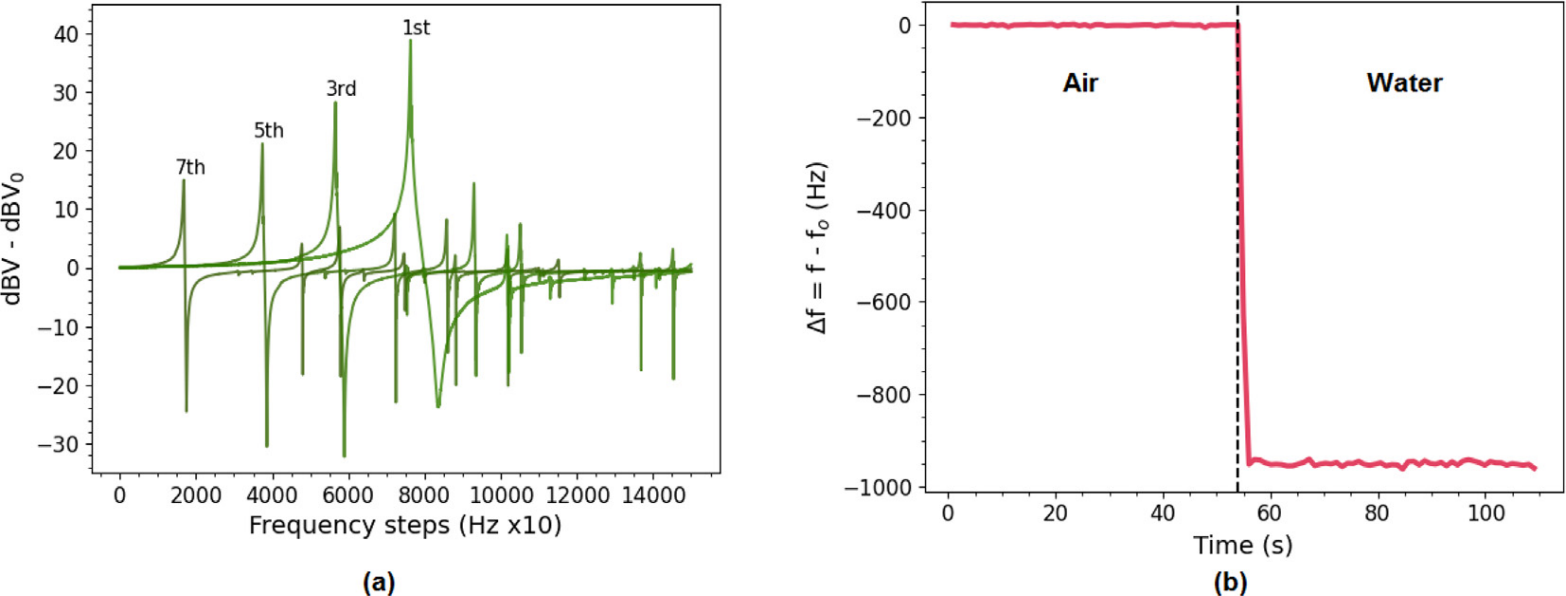
(a) In air measurements of the fundamental resonance frequency, 3rd, 5th and 7th overtone of a 5 MHz crystal in air from Microvacuum showing its much stronger fundamental resonance than the OpenQCM crystals. The 9th and 11th overtone are omitted as they fell outside of the plot area. It is also important to note that for the Microvacuum crystal the fundamental overtone is the dominant overtone, whereas for the OpenQCM crystal this was not the case. (b) Frequency measurement of OpenQCM crystal upon water addition showing a shift greater than predicted by Kanazawa-Gordon (∼750 Hz).

Measurements of the resonance response of a 5 MHz AT-cut quartz crystal in air and liquid were used to assess the capability of the EQCM-I to excite the fundamental frequency and its harmonics. The results can be seen in Fig. 13 which shows a clear fundamental and harmonic resonance features belonging to the quartz up to the 11^*th*^ overtone. Spurious features likely caused by parasitics can also be distinguished, but are always attenuated relative to the main resonance. Interestingly, Fig. 13a shows that the fundamental resonance appears to be weaker than the third overtone in air, which is contrary to what would be expected. This trend was also observed for other crystals of this brand, while a test using a different brand did reveal a stronger fundamental resonance compared to its overtones (Fig. 14a). Therefore, it is likely that damping of the fundamental overtone is caused by the quartz crystal quality and not the device. On the other hand, Fig. 13b shows that in liquid the fundamental overtone is now the dominant signal and that the magnitude of the signal decays with higher overtones as expected. Furthermore, it can be observed that the in-liquid resonances are significantly damped with respect to their in-air analog. This is caused by the viscous loading of the crystal as was previously explained in Section 1.2.1. While the device in principle allows for measurements up to 70 MHz the signal amplitude starts to decay rapidly after the 5^th^ overtone in liquid. The decreased amplitude together with the 12-bit resolution of the ADC complicate a high resolution frequency measurement. To ascertain an appreciable resolution of around 1 Hz in liquid oversampling of the ADC is employed. This however impacts the time resolution of the measurements and complicates simultaneous measurements at higher overtones. A possible solution to overcome these limitations could include the use of a high speed, high resolution ADC. However, currently that is out of the scope of this paper as it is meant to present the first step towards an open-source EQCM-I.

A further experiment that supports the idea that the quartz crystal quality is not optimal is shown in Fig. 14b. Here the shift in resonance frequency as predicted by the Kanazawa-Gordon equation when switching from air to liquid water is demonstrated. However, the shift is larger than the expected value of 750 Hz for water. This could be due to a high surface roughness of the electrodes, which ideally should be as smooth as possible.

**Fig. 15:**
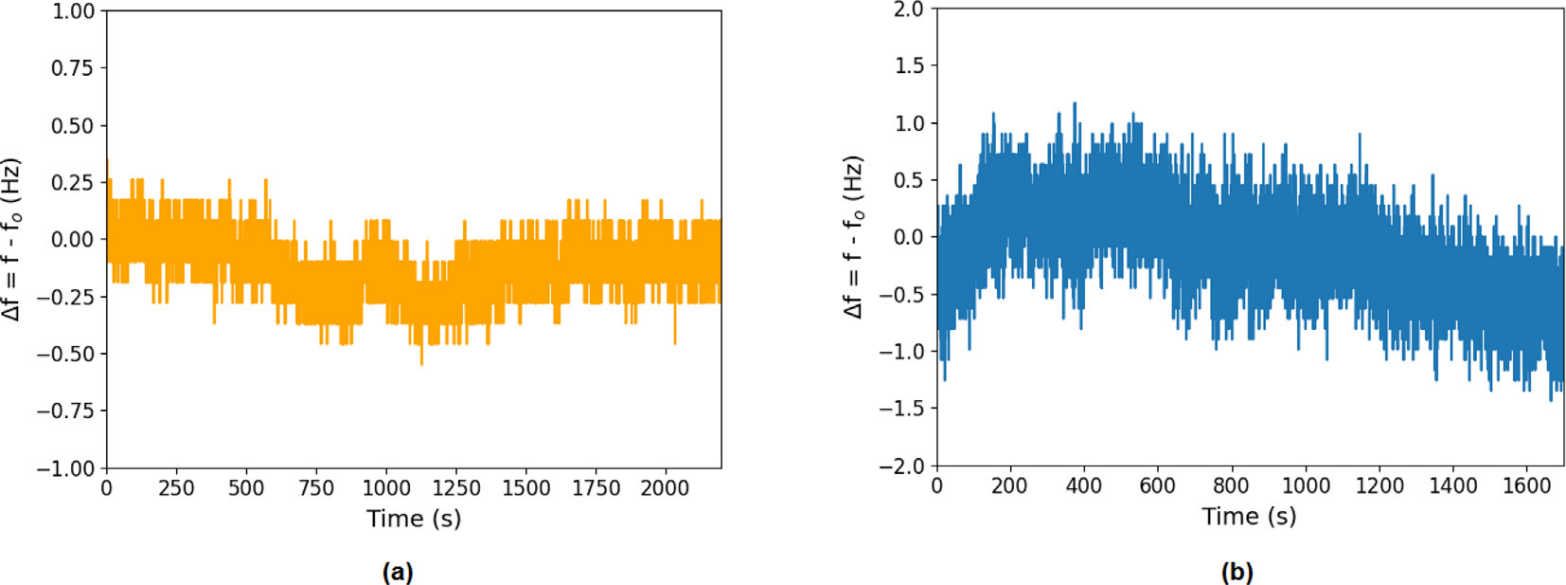
(a) Shows a measurement to test the resonance frequency stability of an OpenQCM crystal in air over a prolonged period of time. (b) Shows the same kind of test for this crystal in a liquid (water) environment.

In an effort to characterize the performance of the device in terms of accuracy drift tests were performed. The results can be seen in Fig. 15a,b for a measurement in air and water respectively. It can be observed that the measurement in air has a superior stability compared to measuring in liquid. In air the measured value for $f_{s}$ varies ±0.25 Hz while the stability of the measurement in water deviates ±0.6 Hz. This can be explained by the decreased resonance frequency sensitivity because of liquid damping (lower quality factor) of the admittance measurement. For air and water this deviation translates to an error bar of roughly ±4.4 ng cm^−2^ and ±10.6ng cm^−2^. It can further be seen that there is a slight drift in resonance frequency over time, but this could be related to slight changes in temperature as the resonance frequency is quite sensitive to temperature changes. This shows that temperature control is important especially for lengthy experiments and processes where heat is generated.

**Fig. 16:**
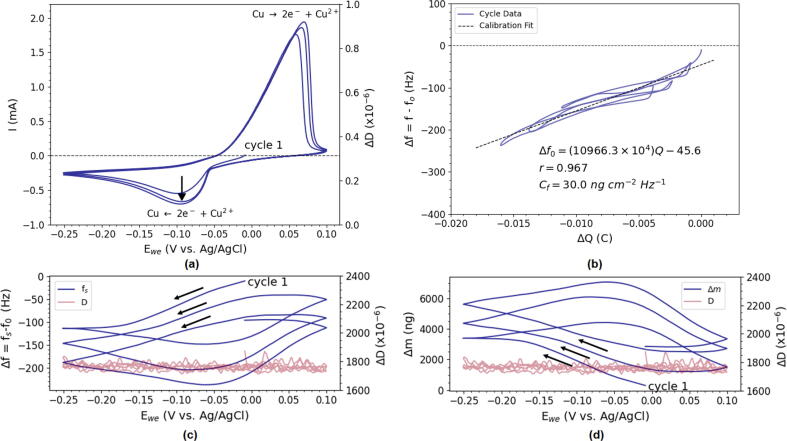
Cyclic voltammogram of Cu-electrodeposition from a 5 mM CuSO_4_ + 0.5 M Na_2_SO_4_ solution between −0.25 and  + 0.1 V vs. Ag/AgCl at 10 mVs^−1^ (a). Simultaneous EQCM-I measurements (c,d) of the 5 MHz fundamental frequency showing Δf and Δm with respect to the WE-potential (blue) and the measured dissipation (red). Graph of Δf vs. ΔQ for the determination of the calibration constant, $C_{f}$. Insets show linear regression results.

To further illustrate the potential of the device it was tested in electrodeposition of copper using a 5 mM CuSO_4_ + 0.5M Na_2_SO_4_ solution. The results can be seen in Fig. 16 as the cyclic voltammogram (CV) shows two distinct features related to the oxidation and reduction of Cu. The graph below shows the corresponding frequency and dissipation response upon deposition and partial dissolution. The reductive current in the CV corresponds to a decrease in the resonance frequency as would be expected from Cu-deposition, whereas the oxidative current is accompanied by a frequency increase signalling mass loss.

It can further be seen that deposition and dissolution of Cu has no significant impact on the dissipation which rests steadily at ΔD = 1.8x10^−4^. This is to be expected from a rigid metallic layer as explained in Section 1.2.1. A further study into the relation between cumulative charge (ΔQ) and Δf, which allows for the calibration of the EQCM as explained in Section 1.2.2, can be seen in Fig. 16c. The calculated $C_{f}$ deviates significantly (>40%) from the theoretical value, likely caused by competition from the oxygen reduction reaction (ORR) as O_2_ could not be excluded in this particular experiment.

Aside from the capability of measuring adsorption of a rigid Cu layer it is also important to assess the device in a situation where dissipation monitoring is important. For this assessment the oxidative electropolymerization of aniline was used as it is a well studied system that shows well-defined electrochemistry and vivid electrochromicity [20]. During potential cycling in an acidic solution of aniline a layer of polyaniline (PANI) is deposited each cycle in the oxidative regime. For this 10 mM aniline in 1M of HClO_4_ was cycled 25 times in a potential range of −0.2 V to 0.9 V vs. Ag/AgCl (3M NaCl) at 50 mVs^−1^. Prior to the CV the potential was equillibrated at 0.1 V vs. Ag/AgCl for 2 s.

**Fig. 17:**
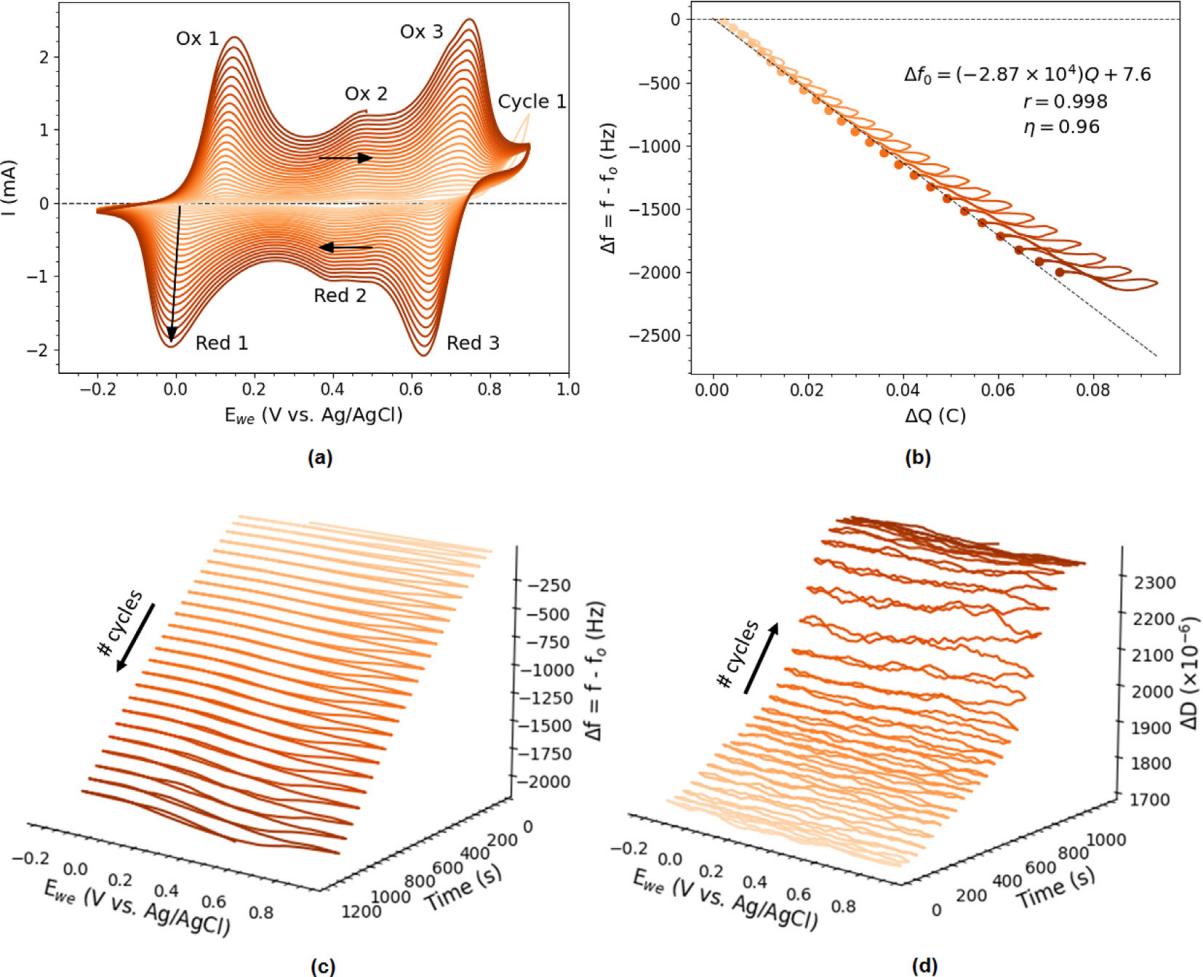
(a) CV of the oxidative electropolymerization of aniline monitored by EQCM-I (25 cycles, −0.2 V to 0.9 V at 50 mVs^−1^ in solution of 10 mM aniline in 1M of HClO_4_). (b) Plot of ΔQ vs. Δf with fit inset used to calculate the Faradaic efficiency of polymerization (η). (c) & (d) plots of the frequency and dissipation as a function of time and potential during the electropolymerization.

**Fig. 18:**
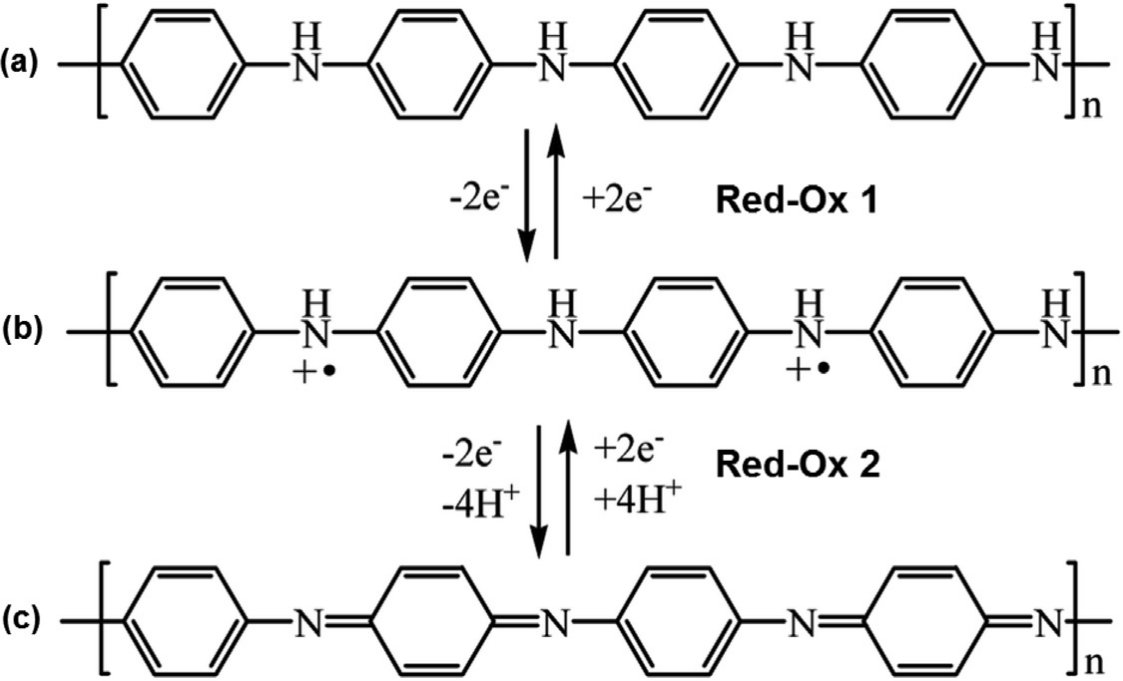
Chemical representation of the reduced leucoemeraldine (a), which is oxidized to emeraldine (b) and further oxidized to pernigraniline possessing imine functionalities. Reproduced from Adriianova et al. [42].

The resulting CV can be seen in Fig. 17a and it exhibits three characteristic redox features. At 0.78 V aniline is oxidized and the deposition of PANI takes place as could be concluded from frequency decrease in the EQCM-I measurements (Fig. 17b,c). The intensity of the three redox features increases per cycle and so does the magnitude of the negative frequency shift. Inspection by eye reveals that between Red-Ox 1 and Red-Ox 3 the color changes from a translucent yellow to green to dark purple. This is known to be the change from leucoemeraldine to emeraldine and to pernigraniline phase of PANI. Red-Ox 2 is often attributed to side reactions [20]. A schematic representation of the PANI phase changes is given in Fig. 18. The difference between leucoemeraldine and emeraldine is said to be the partial oxidation of the PANI backbone and incorporation of anion dopants (ClO_4_^−^) [20]. This is evidenced by the fact that Δf shows a certain hysteresis. At −0.2 V all the dopants are expelled from the layer and just the pure PANI backbone is measured, while a clear negative shift in resonance frequency is observed at higher potentials. Subtraction of the polymer backbone shift from the most negative shift of a cycle can reveal the mass associated with anion dopants [41].

### 7.4 Summary

The goal of this work was to design and test an open-source and affordable EQCM-I capable of measuring all relevant parameters a commercial EQCM could assess. The impedance-based design was built on the foundations of the OpenQCM project and improvements were made in terms of signal integrity at high frequencies, amplitude control, faster and ∼30 dB more sensitive measurements. The device was demonstrated to be able to excite 5 MHz quartz crystals up to the 11^th^ overtone in air and liquid.

The applicability of the device to electrochemical research was validated based on two electrochemical systems. The first involving Cu-electrodeposition to successfully showcase the combination of electrochemistry with the impedance-based quartz crystal microbalance measurements. As expected the metal deposition could be actuated and monitored simultaneously by both techniques, with a clear electrochemical response and shift in resonance frequency while the dissipation remained unchanged. The second system concerned the oxidative electropolymerization of aniline which the device was also able to characterize successfully. Surprisingly, it was even able to pick up the slight change in dissipation which would be expected upon the formation of the thin viscoelastic polymeric film.

Further device improvements are still needed to permit simultaneous measurements at higher overtones to be able to also be applicable to complex viscoelastic systems. Especially in liquid, the quartz resonance at higher harmonics suffered from strong viscous damping and this complicated measurement at sufficient frequency and time resolution. Overall, the device has shown that it is sufficiently capable of measuring all the parameters relevant to an EQCM-I. Nevertheless, some opportunities are identified to further improve the performance of the device which could potentially put its next generation on par with commercial systems.

### 7.5 Outlook

It should have become clear that the device is capable of measuring all the parameters relevant to an EQCM-I, but still suffers from limitations related to frequency and time resolution. While technically possible, the ambition to measure in liquid at various overtones simultaneously proved difficult due to the decrease in acquisition speed it would entail. The extensive ADC conversion averaging required to increase the signal-to-noise ratio negatively impacts acquisition time. To overcome these limitations improvements are suggested based on the software, hardware and measurement setup.

While the DAQ and MCU code worked to perform the intended measurements it is highly conceivable that the separate programs can be further optimized. There are various approaches to performing a frequency sweep code-wise. Currently, the 4096 ADC averages per frequency measurement is the most time-intensive operation (>4 million ADC conversions per sweep). One option would be to use a non-linear sweep with low resolution over the wide frequency range to still be able to capture the admittance plot, while still keeping a high frequency resolution around $f_{s}$ so as not to compromise resolution for speed. Another area of improvement would be the replacement of the Python DAQ script by a faster programming language like C as the DAQ script execution also slows down the measurements. This however would come at the cost of a less beginner-friendly programming environment.

In terms of hardware a couple of changes could also be made that could potentially benefit the operation of the device. As mentioned in the software recommendations the numerous ADC conversions form the bottleneck of the high resolution acquisition. The Teensy 4.0 features two 12-bit (effectively 10-bit without averaging) ADCs which are not specifically designed for high resolution measurement devices. Therefore, it might be an option to fit the EQCM-I with an additional ADC chip with higher bit resolution or sampling rate to perhaps reduce the amount of averaging needed or increase the amount of samples measured per second and thereby decreasing acquisition time. This will however make the design more complex, since the communication with an external ADC can be challenging. Another option would be to switch to the faster QCM-D measurement principle, where lengthy frequency sweeps are not required. For this change the board would essentially only need a programmable switch to perform the ring-down measurement as all the other components should be able to work in a QCM-D type approach. Additionally, the signal gain control, currently set by the AD5252-controlled current limit, could be handled by the AD8007 opamp so that signal amplitudes outside of the AD9851 DAC limits could be achieved. This could help in gaining improved resolution at higher overtones in liquid. It must be noted that achieving gain control through the opamp will be challenging considering the high signal frequency.

Finally, the cell used for the measurements did not allow for proper temperature control and shielding of the signals and therefore caused quite some noise issues. A custom shielded cell could significantly benefit the measurement stability as the board already possesses the necessary shielded twin-axial connectors.

## CRediT authorship contribution statement

**Rens J. Horst:** Conceptualization, Methodology, Software, Validation, Investigation, Visualization, Writing - original draft, Writing - review & editing. **Antonis Katzourakis:** Conceptualization, Software. **Bastian T. Mei:** Writing - review & editing, Supervision. **Sissi de Beer:** Writing - review & editing, Supervision.

## Declaration of Competing Interest

The authors declare that they have no known competing financial interests or personal relationships that could have appeared to influence the work reported in this paper.
